# Tinnitus-Related Distress and the Personality Characteristic Resilience

**DOI:** 10.1155/2014/370307

**Published:** 2014-07-08

**Authors:** Elisabeth Wallhäusser-Franke, Wolfgang Delb, Tobias Balkenhol, Wolfgang Hiller, Karl Hörmann

**Affiliations:** ^1^Medical Faculty Mannheim, Heidelberg University, ENT-Department, Unit of Phoniatrics and Audiology, Ludolf-Krehl-Straße 13-17, 68167 Mannheim, Germany; ^2^HNO-Kooperation Südwestpfalz, Pfaffplatz 10, 67659 Kaiserslautern, Germany; ^3^Department of Psychology, Johannes Gutenberg University of Mainz, Wallstraße 3, 55122 Mainz, Germany; ^4^University Medical Centre Mannheim, ENT-Department,Theodor-Kutzer-Ufer 1-3, 68167 Mannheim, Germany

## Abstract

It has been suggested that personality traits may be prognostic for the severity of suffering from tinnitus. Resilience as measured with the Wagnild and Young resilience scale represents a positive personality characteristic that promotes adaptation to adverse life conditions including chronic health conditions. Aim of the study was to explore the relation between resilience and tinnitus severity. In a cross-sectional study with a self-report questionnaire, information on tinnitus-related distress and subjective tinnitus loudness was recorded together with the personality characteristic resilience and emotional health, a measure generated from depression, anxiety, and somatic symptom severity scales. Data from 4705 individuals with tinnitus indicate that tinnitus-related distress and to a lesser extent the experienced loudness of the tinnitus show an inverse correlation with resilience. A mediation analysis revealed that the relationship between resilience and tinnitus-related distress is mediated by emotional health. This indirect effect indicates that high resilience is associated with better emotional health or less depression, anxiety, and somatic symptom severity, which in turn is associated with a less distressing tinnitus. Validity of resilience as a predictor for tinnitus-related distress is supported but needs to be explored further in longitudinal studies including acute tinnitus patients.

## 1. Introduction

Subjective tinnitus, an internal sound generated by aberrant activation within the auditory system, is a widespread phenomenon which constitutes a severe problem for 10%–20% of the tinnitus population [1]. The distress associated with tinnitus shows closer relation with factors related to emotional health as depression, anxiety, and somatic symptom severity than with the loudness of the tinnitus [2]. Moreover, depression and anxiety were found to be enhanced at tinnitus onset in patients who later develop high tinnitus-related distress [3, 4] suggesting that emotional health may be prognostic for future tinnitus-related distress. It cannot be excluded, however, that distressing tinnitus adversely influences emotional health and that its association with depression and anxiety is overestimated due to content overlap in the questionnaires [5, 6]. Therefore, alternative predictors, which are largely independent of the actual tinnitus-related distress, are needed for the prognosis of future tinnitus-related distress.

Personality characteristics display continuity throughout life, they have a predictive role for mental and physical health, and content overlap with the tinnitus questionnaires is not an issue. Personality characteristics associated with distressing tinnitus are emotional lability indicated by increased neuroticism and decreased extraversion [7–10] and the tendency to experience fear when perceiving body signs of arousal (rev. in [11]). Consequently, trait anxiety correlates with tinnitus-related distress, and distressed type D personality is overrepresented in tinnitus populations (rev. in [11, 12]). Also, significant numbers of somatic symptoms, which are linked to the personality traits of neuroticism or negative affectivity [13], are found in a substantial portion of tinnitus patients [2], and depression and anxiety decreased with time only in those tinnitus patients that did not exhibit personality disorders [9]. Although there is no consensus about the role of personality for tinnitus severity [8], personality may influence the way tinnitus is dealt with and especially influence the persistence of tinnitus through a personality-driven tendency to be aware of it [9].

The concept of the positive personality characteristic resilience delineates capabilities of an individual to cope effectively with adverse life conditions such as chronic disease [16, 17]. The personality traits emotional stability and extraversion are associated with resilience [18], whereas depression and anxiety are inversely related to it [19]. Resilience was linked to psychobiological mechanisms that keep the hypothalamic-pituitary-adrenal (HPA) axis and the noradrenergic system, which are suspected to promote tinnitus-related distress [20] within an optimal range during stress exposure and terminate the stress response early [21, 22]. This is thought to be largely determined by genetic disposition in conjunction with early life experiences [23, 24].

The Wagnild and Young resilience scale was shown to be an appropriate instrument to study the personality characteristic resilience in adult populations (rev. in [16, 23–28]), and short versions of this scale are increasingly being used [25, 26, 29–31]. The German short version (RS-13) has been validated in representative clinical and nonclinical samples [25, 26].

Aims of the present study were to assess trait aspects of resilience in a tinnitus population and to relate these to measures of tinnitus-related distress and subjective tinnitus loudness. To gain an understanding for causal relationships between the personality characteristic resilience, the emotional health measures depression, anxiety, and somatic symptom severity, and the tinnitus-associated symptoms tinnitus-related distress and tinnitus loudness, we established a mediator model [32]. We hypothesized that the personality characteristic resilience is an important factor for determining the reaction on tinnitus as reflected in the amount of tinnitus-related distress, and that much of its influence is conveyed through a factor emotional health generated from current status of depression, anxiety, and somatic symptom severity. Furthermore, we hypothesized that the influence of resilience on tinnitus-related distress is higher than its influence on the subjectively perceived loudness of the tinnitus, which is thought to be affected primarily by hearing-related pathologies [2].

## 2. Methods 

### 2.1. Data Collection and Sample

A questionnaire was sent to all 13,349 patient members of the German Tinnitus Association (DTL) together with a letter informing the participants that by filling out and sending in the questionnaire they agreed to the use of their data for research purposes. The DTL is a registered charity that provides information, support, and advice about tinnitus and funds research thereby aiming to raise awareness about the condition. 4752 questionnaires (35.6%) were received, and the data of 4705 questionnaires were entered into the data base. The rest was omitted mainly because of invalid membership numbers [2]. Questionnaires were pseudonymised in that they contained the membership code but not the participants' names. The study protocol was approved by the ethics committee and by the data safety commissioner of the Medical Faculty Mannheim of Heidelberg University according to the principles expressed in the Declaration of Helsinki. The following parts of the questionnaire were used.

### 2.2. Measures

Tinnitus-related distress was assessed with the 12-item Mini-Tinnitus Questionnaire (MTQ [14]). The MTQ represents an abridged version of the Tinnitus Questionnaire. It defines a general dimension of distress that has a high degree of correlation (*r* = .90) with the full Tinnitus Questionnaire. The test-retest reliability of the MTQ was .89 [14]. Sum scores range from 0 (no distress) to 24 (maximum distress) and were derived only from cases with complete MTQ-scales. Subjectively perceived tinnitus loudness was recorded on a numeric rating scale (T-NRS) from 0 (tinnitus audible only during silence) to 10 (tinnitus louder than all external sounds).

Resilience was addressed with the RS13 questionnaire [26]. The RS13 has a high correlation with the longer 25-item form of the resilience scale (*r* = .95), and its internal consistency is high with a Cronbach's alpha of .91 [26]. Response options ranged from 1 (strongly disagree) to 7 (strongly agree). A sum score was calculated from the 13 items with scores between 13 and 91, and higher scores indicating better resilience.

In addition three modules of the Patient Health Questionnaire (PHQ) addressing depression (PHQ9), generalized anxiety (GAD7), and somatic symptom severity (PHQ15) were included [15, 33]. These PHQ-scales have been used in clinical studies across a variety of medical conditions; their internal consistency is high with a Cronbach's alpha of .8 or above for PHQ15 and PHQ9 and a test-retest reliability around .83 for the three scales [33]. Response options for PHQ9 and GAD7 were 0 (not bothered at all) to 3 (bothered almost every day), and for PHQ15 they were 0 (not bothered at all) to 2 (bothered a lot). In all PHQ modules higher scores indicated greater symptom severity [15]. A case was eliminated for classification in a scale if a single item was missing, but if the two items addressing premenopausal women and sexually active persons in PHQ15 were left blank, they were scored as 0 [2].

### 2.3. Data Analysis

Analyses were performed with SPSS22. Bivariate and partial correlation coefficients were calculated to verify relations among the variables. Because correlation between the variables depression, anxiety, and somatic symptom severity were high, a variable “emotional health” (EH) was generated from the *z*-standardized PHQ-scales. Low values in the EH variable represent the more favourable condition of better emotional health. For the following analyses the variables RS13, MTQ, and T-NRS were *z*-standardized as well. Two stepwise regression analyses quantified the extent to which EH and RS-13 explained variance in tinnitus-related distress and subjective tinnitus loudness. Finally, direct and indirect effects of the personality characteristic resilience on tinnitus-related distress were assessed in a mediation analysis, using the SPSS macro provided by [31]. In this model, EH which was significantly correlated with both RS13 and MTQ was considered to be a potential mediator between the personality trait resilience and tinnitus-related distress. Causal order of the variables with the personality characteristic resilience as independent variable, emotional health as mediator, and tinnitus-related distress as outcome was based on theoretical grounds. As recommended by [32], significance of the indirect effect was also tested by means of a bootstrap analysis with 5000 bootstrap samples.

## 3. Results

### 3.1. Sample Characteristics and Bivariate Correlations

The sample has been described in detail in a preceding publication [2]. Resilience had been recorded along with the other variables but was not included in the previous analysis [2]. 4705 participants provided their data; 59.1% of them were male. Since results did not deviate substantially between genders (see Table 1), results are reported for the whole sample. Mean age was 58.6 [SD = 11.8] and 84% experienced tinnitus for more than 5 years. With a mean of 10.4 [6.5] the average sum score of the MTQ (Table 1) fell into the category of moderate tinnitus-related distress, with 37.6% reporting mild distress (MTQ ≤ 7), whereas 13.4% felt severely distressed by their tinnitus (MTQ ≥ 19). Cronbach's alpha for MTQ was high in this sample with 0.91, as well as for the three PHQ-scales with .87 for PHQ9; .90 for GAD7; and .81 for PHQ15. In the PHQ scales a score of 10 and above is the most commonly recommended cut point for clinically significant symptoms on all three scales [33]. Averages for each of the scales were below 10 (Table 1), but 20.6%, 27%, and 35.8% reached scores of 10 or above in the PHQ9, GAD7, or PHQ15, respectively. In contrast to the other scales, higher scores in the RS13 scale are desirable. Average of RS13 was 66.4 [15.1] (Table 1) which is slightly lower than that found in a normative sample ([26]: 70.0 [9.0]). Again, Cronbach's alpha for RS13 was high with .93.

### 3.2. Bivariate Correlations and Regression Analyses

All bivariate correlations were highly significant. The highest correlations were observed among the PHQ variables (Table 2). For tinnitus-related distress correlations were higher with depression and anxiety than with the subjectively perceived tinnitus loudness or somatic symptom severity. Inverse relations existed between all these variables and the RS13 resilience scale. Correlations of RS13 with the three PHQ-scales were higher than with tinnitus-related distress. Thus higher or more positive resilience scores were linked to lower levels of depression, anxiety, and somatic symptom severity as well as to lower tinnitus-related distress. All correlations with subjective tinnitus loudness were conspicuously lower, and the lowest was the inverse correlation between T-NRS and RS13 (Table 2). Subsequently, two stepwise regression analyses were performed, one with MTQ and the other with T-NRS as dependent variable, to quantify the extent to which the PHQ-measures and RS13 explain variance in tinnitus-related distress and subjective tinnitus loudness, respectively. For these analyses, the three PHQ-scales were comprised into the variable emotional health (EH). Since the PHQ-scales have different ranges (Table 1), the variables were *z*-standardized prior to averaging. In addition, the other variables included in the regression analyses were *z*-standardized as well. Results of the regression analysis with MTQ as dependent variable evidenced that EH contributed 43.3% to the total of 43.4% of the explained variance in MTQ, while the influence of RS13 on MTQ was negligible (Table 3(a)). The second regression analysis with T-NRS as dependent variable showed that EH and RS13 only explained about 12% of the variance in T-NRS, and again the effect of RS13 was negligible (Table 3(b)).

### 3.3. Indirect Effect of Resilience on Tinnitus-Related Distress

Finally, a mediation analysis was conducted with the *z*-standardized values of the variables MTQ, RS13, and EH. For this analysis RS13 served as independent variable, EH served as mediator, and MTQ was the dependent variable. Results of this analysis were in line with the assumption that resilience has a significant, although indirect, effect on tinnitus-related distress. The total effect of RS13 on MTQ expressed as *β* was −.399. Most of this effect was indirect (*β* = −.360) and in the model was conveyed via the mediator variable EH. The direct effect of resilience on tinnitus-related distress was of much smaller magnitude with a *β* of −.038. Moreover, whereas the direct effect barely reached significance with *P* = .048, the total and the indirect effects of RS13 on MTQ through the mediator EH were significant (Table 3(c)).

## 4. Discussion

To the best of our knowledge this is the first study to explore the relation of the positive personality characteristic resilience with tinnitus-related distress and subjective tinnitus loudness in a large tinnitus population. Results of a bivariate analysis indicate that the correlations of resilience and of emotional health (a factor generated from depression, anxiety, and somatic symptom severity scores) with tinnitus-related distress are higher than with perceived tinnitus loudness confirming the distinction between these tinnitus characteristics reported earlier [14]. Results of the bivariate analysis furthermore indicate a significant correlation between resilience and emotional health corroborating earlier findings in population samples that were selected for characteristics other than tinnitus [25, 34]. Results of a regression analysis that considers resilience and emotional health in conjunction indicate that current emotional health has a large effect on tinnitus-related distress but a small effect on subjective tinnitus loudness, whereas resilience has a negligible effect on both tinnitus characteristics. Finally, results of a mediator analysis which serves to reveal indirect effects of a factor on an outcome variable are in line with the interpretation that resilience has an indirect effect on tinnitus-related distress conveyed by the present status of emotional health. As the personality trait resilience is fairly stable throughout life [16, 23, 24] while tinnitus usually arises at middle or older age [2], low resilience is unlikely to develop as a result of current low emotional health or through experiencing distressing tinnitus. Rather, low resilience may promote an unfavourable emotional health status which in turn may promote high tinnitus-related distress. Along this line of reasoning, the study extends prior research on an association between personality and tinnitus characteristics by suggesting that personality has an indirect influence on tinnitus severity conveyed via general emotional health.

Resilience is a personality characteristic associated with adaptation to adverse chronic health conditions. Individuals with high resilience scores exhibit emotional stability and possess a behavioural repertoire that allows them to face stress and adversity in such a way that they retain their emotional balance. High resilience has been associated with an internal locus of control [35], that is, the extent to which an individual perceives an event to be under his own control, and an internal locus of control was found to be associated with lower tinnitus-related distress [36]. Usually highly distressed tinnitus patients believe that they cannot influence their tinnitus (external locus of control) and as a consequence they do not apply effective coping strategies [36, 37]. Interestingly, a mediating effect of coping on the relation between illness representations and adjustment to the tinnitus has been reported recently [38].

Although results have to be interpreted within the limits of a cross-sectional design, they are consistent with the interpretation that resilience has an indirect effect on tinnitus severity which is mediated by current emotional health. This interpretation is corroborated by longitudinal studies, which suggest that depression and anxiety levels at tinnitus onset are related to the progression of tinnitus-related distress [3, 4]. Furthermore, it was observed that depression and anxiety in tinnitus sufferers decreased with time only in those tinnitus patients that did not exhibit personality disorders [9]. Even though ultimate proof for the validity of these interactions requires further prospective studies, testable interactions between the variables are suggested.

Some other limitations of the present study should be noted. As the members of the DTL are a self-selected sample, they may not be representative of the general tinnitus population. The distribution of resilience in the study sample is comparable to that of other studies with the same instrument, however [26, 27]. Furthermore, it cannot be excluded that some questions of the self-report questionnaire were misunderstood or were reported incorrectly. The resilience scale does not contain items to control for response biases. Though high consistency within the scale as indicated by a high Cronbach's alpha as well as the distribution of resilience in the study sample which is comparable to that of other studies with the same instrument, together with data obtained with other personality inventories [39, 40], argue against intentional bias in tinnitus populations.

## 5. Conclusions

Analysing data from a large tinnitus population we found that low resilience is associated with low emotional health and with distressing tinnitus. When considering the personality trait resilience and the current status of emotional health in conjunction, resilience has only a minor effect on tinnitus characteristics. Because of its association with emotional health, resilience may nevertheless serve as an indicator for future development of tinnitus-related distress, since it is less likely to be influenced by adverse transient life conditions and by distressing tinnitus than emotional health. This needs to be verified in longitudinal studies involving patients with acute tinnitus.

## Figures and Tables

**Table 1:** Descriptive statistics.

	Number of valid answers	Mean [SD] or %	Q1–median–Q3	Range	Female//male mean [SD] or %
Male	4606	59.1			
Age	4490	58.6 [11.8]	50–59–68	18–94	57.4 [12.2]//59.5 [11.4]
Tinnitus duration >5 years	4608	84.0			81.1//87.7
Tinnitus-related distress (MTQ)	4661	10.4 [6.5]	5–10–15	0–24	10.3 [6.2]//10.5 [6.5]
Subjective tinnitus loudness (T-NRS)	4372	6.0 [2.5]	4–6–8	0–10	5.9 [2.5]//6 [2.5]
Depression (PHQ9)	4369	7.1 [5.4]	3–6–10	0–27	7.5 [5.2]//6.9 [5.5]
Anxiety (GAD7)	4546	6.0 [4.8]	3–5–8	0–21	6.4 [4.8]//5.7 [4.8]
Somatic symptom severity (PHQ15)	4131	8.4 [5.2]	4–7–11	0–32	9.4 [5.3]//7.7 [5.1]∗∗
Resilience (RS13)	4396	66.4 [15.1]	57–69–78	13–91	65 [15.1]//67 [15]

Demographic, psychological, and tinnitus characteristics of the study sample. Gender differences were minor, except for the somatic symptom scale PHQ15 (∗∗), in which females could reach higher scores than males (see Section 2).

**Table 2:** Bivariate correlations.

	MTQ *r* (95% CI)	T-NRS *r* (95% CI)	RS13 *r* (95% CI)	PHQ9 *r* (95% CI)	GAD7 *r* (95% CI)
MTQ	1				
T-NRS	.526 [.498–−.551]∗∗	1			
Resilience (RS13)	−.399 [−.428–−.369]∗∗	−.132 [−.165–−.098]∗∗	1		
Depression (PHQ9)	.667 [.646–.687]∗∗	.352 [.322–.382]∗∗	−.559 [−.584–−.533]∗∗	1	
Anxiety (GAD7)	.616 [.593–.637]∗∗	.303 [.271–.333]∗∗	−.548 [−.574–−.523]∗∗	.805 [.790–.819]∗∗	1
Somatic symptom Severity (PHQ15)	.540 [.514–.564]∗∗	.303 [.271–.333]∗∗	−.440 [−.468–−.413]∗∗	.758 [.742–.773]∗∗	.655 [.634–.675]∗∗

Bivariate Spearman-Rho correlation coefficients and their 95% confidence limits (95% CI) are reported. Confidence limits that do not include 0 are considered significant. MTQ—tinnitus-related distress assessed with the 12-item Mini Tinnitus Questionnaire [14], T-NRS—tinnitus loudness rated on a numeric rating scale. **P < .001.

**(a):** 

Independent variables	*β* Step 1	*β* Step 2
Step 1. adj. R^2^ = .433, F(1, 4327) = 3301.43∗∗∗		
Emotional health	.658∗∗∗	
Step 2. adj. R^2^ = .434, ΔF(2, 4326) = 7.97∗∗		
Emotional health		.636∗∗∗
Resilience		−.039∗∗

**(b):** 

Independent variables	*β* Step 1	*β* Step 2
Step 1. adj. *R* ^2^ = .119, *F*(1, 4092) = 552.65∗∗∗		
Emotional health	.345∗∗∗	
Step 2. adj. *R* ^2^ = .123, Δ*F*(2, 4091) = 21.05∗∗		
Emotional health		.391∗∗∗
Resilience		.081∗∗∗

**(c):** 

Effect	*β*	BCa 95%	
		Lower	Upper
IV (RS13)—mediator (EH)	−.614∗∗∗		
Mediator (EH)—DV (MTQ)	.586∗∗∗		
IV—DV direct effect	−.038∗		
IV—DV indirect effect	−.360	−.385	−.324
IV—DV total effect	−.399∗∗∗		
Adj. *R* ^2^ = .434, *F*(2, 4326) = 1657.36∗∗∗			

(a) A stepwise regression analysis with the *z*-standardized variables emotional health (EH) and resilience (RS13) as independent and tinnitus-related distress (MTQ) as dependent variable.

(b) A stepwise regression analysis with the *z*-standardized variables emotional health (EH) and resilience (RS13) as independent and subjective tinnitus loudness (T-NRS) as dependent variable.

(c) Mediation was subsequently tested with *z*-standardized RS13 as independent (IV) and *z*-standardized MTQ as dependent (DV) variable and the *z*-standardized variable EH as mediator. The mediation effects were estimated by bootstrap analyses [15].

BCa 95% CI = bias corrected 95% confidence interval based on 5000 bootstrap samples.

A confidence interval that does not contain 0 indicates a significant effect.

***P < 0.001, **P < 0.01, and *P < 0.05.

## References

[1] Krog NH, Engdahl B, Tambs K (2010). The association between tinnitus and mental health in a general population sample: results from the HUNT Study. *Journal of Psychosomatic Research*.

[2] Wallhäusser-Franke E, Brade J, Balkenhol T, D'Amelio R, Seegmüller A, Delb W (2012). Tinnitus: distinguishing between subjectively perceived loudness and tinnitus-related distress. *PLoS ONE*.

[3] D'Amelio R, Archonti C, Scholz S, Falkai P, Plinkert PK, Delb W (2004). Psychological distress associated with acute tinnitus. *HNO*.

[4] Langenbach M, Olderog M, Michel O, Albus C, Köhle K (2005). Psychosocial and personality predictors of tinnitus-related distress. *General Hospital Psychiatry*.

[5] Ooms E, Meganck R, Vanheule S, Vinck B, Watelet J-B, Dhooge I (2011). Tinnitus severity and the relation to depressive symptoms: a critical study. *Otolaryngology—Head and Neck Surgery*.

[6] Ooms E, Vanheule S, Meganck R, Vinck B, Watelet J, Dhooge I (2012). Tinnitus severity and its association with cognitive and somatic anxiety: a critical study. *European Archives of Oto-Rhino-Laryngology*.

[7] Langguth B, Kleinjung T, Fischer B, Hajak G, Eichhammer P, Sand PG (2007). Tinnitus severity, depression, and the big five personality traits. *Progress in Brain Research*.

[8] Tyler RS, Coelho C, Noble W (2006). Tinnitus: standard of care, personality differences, genetic factors. *ORL*.

[9] Welch D, Dawes PJD (2008). Personality and perception of tinnitus. *Ear and Hearing*.

[10] McCormack A, Edmondson-Jones M, Fortnum H (2014). The prevalence of tinnitus and the relationship with neuroticism in a middle-aged UK population. *Journal of Psychosomatic Research*.

[11] Malouff JM, Schutte NS, Zucker LA (2011). Tinnitus-related distress: a review of recent findings. *Current Psychiatry Reports*.

[12] Bartels H, Pedersen SS, Van Der Laan BFAM, Staal MJ, Albers FWJ, Middel B (2009). The impact of type D personality on health-related quality of life in tinnitus patients is mainly mediated by anxiety and depression. *Otology and Neurotology*.

[13] Russo J, Katon W, Sullivan M, Clark M, Buchwald D (1994). Severity of somatization and its relationship to psychiatric disorders and personality. *Psychosomatics*.

[14] Hiller W, Goebel G (2006). Factors influencing tinnitus loudness and annoyance. *Archives of Otolaryngology: Head and Neck Surgery*.

[15] Löwe B, Spitzer RL, Williams JBW, Mussell M, Schellberg D, Kroenke K (2008). Depression, anxiety and somatization in primary care: syndrome overlap and functional impairment. *General Hospital Psychiatry*.

[16] Wagnild G (2009). A review of the resilience Scale. *Journal of Nursing Measurement*.

[17] Ong AD, Bergeman CS, Bisconti TL, Wallace KA (2006). Psychological resilience, positive emotions, and successful adaptation to stress in later life. *Journal of Personality and Social Psychology*.

[18] Sells D, Sledge WH, Wieland M (2009). Cascading crises, resilience and social support within the onset and development of multiple chronic conditions. *Chronic Illness*.

[19] Leppert K, Dye L, Strauss B, Brähler E, Schumacher B, Strauss B (2002). RS—Resilienzskala. *Psychodiagnostische Verfahren in der Psychotherapie*.

[20] Wallhäusser-Franke E, Schredl M, Delb W (2013). Tinnitus and insomnia: Is hyperarousal the common denominator?. *Sleep Medicine Reviews*.

[21] Feder A, Nestler EJ, Charney DS (2009). Psychobiology and molecular genetics of resilience. *Nature Reviews Neuroscience*.

[22] Ozbay F, Fitterling H, Charney D, Southwick S (2008). Social support and resilience to stress across the life span: a neurobiologic framework. *Current Psychiatry Reports*.

[23] Abiola T, Udofia O (2011). Psychometric assessment of the Wagnild and Young's resilience scale in Kano, Nigeria. *BMC Research Notes*.

[24] Choowattanapakorn T, Aléx L, Lundman B, Norberg A, Nygren B (2010). Resilience among women and men aged 60 years and over in Sweden and in Thailand. *Nursing and Health Sciences*.

[25] Leppert K, Strauss B (2011). The role of resilience for coping in different age groups. *Zeitschrift für Gerontologie und Geriatrie*.

[26] Leppert K, Koch B, Brähler E, Strauss B (2008). Resilience scale—evaluation of a long (RS-25) and a short version (RS-13). *Klin Diagnostik Evaluation*.

[27] Portzky M, Wagnild G, de Bacquer D, Audenaert K (2010). Psychometric evaluation of the Dutch Resilience Scale RS-nl on 3265 healthy participants: a confirmation of the association between age and resilience found with the Swedish version. *Scandinavian Journal of Caring Sciences*.

[28] Girtler N, Casari EF, Brugnolo A (2011). Italian validation of the Wagnild and Young Resilience Scale: a perspective to rheumatic diseases. *Clinical and Experimental Rheumatology*.

[29] Damásio BF, Borsa JC, da Silva JP (2011). 14-Item resilience scale (RS-14): psychometric properties of the Brazilian version. *Journal of Nursing Measurement*.

[30] Nishi D, Uehara R, Kondo M, Matsuoka Y (2010). Reliability and validity of the Japanese version of the Resilience Scale and its short version. *BMC Research Notes*.

[31] Perna L, Mielck A, Lacruz ME (2012). Socioeconomic position, resilience, and health behaviour among elderly people. *International Journal of Public Health*.

[32] Preacher KJ, Hayes AF (2008). Asymptotic and resampling strategies for assessing and comparing indirect effects in multiple mediator models. *Behavior Research Methods*.

[33] Kroenke K, Spitzer RL, Williams JBW, Löwe B (2010). The patient health questionnaire somatic, anxiety, and depressive symptom scales: a systematic review. *General Hospital Psychiatry*.

[34] Beasley M, Thompson T, Davidson J (2003). Risilience in response to life stress: the effects of coping style and cognitive hardiness. *Personality and Individual Differences*.

[35] Lundman B, Strandberg G, Eisemann M, Gustafson Y, Brulin C (2007). Psychometric properties of the Swedish version of the Resilience Scale. *Scandinavian Journal of Caring Sciences*.

[36] Delb W, D’Amelio R, Schonecke OW, Iro H Are there psychological or audiological parameters determining tinnitus impact?.

[37] Osterhausen VK, Keßler B, D'Amelio R, Delb W (2001). Maladaptive coping strategies in help-seeking Patients with chronic decompensated tinnitus. *Verhaltenstherapie & Verhaltensmedizin*.

[38] Vollmann M, Kalkouskaya N, Langguth B, Scharloo M (2012). When the ringing in the ears gets unbearable: illness representations, self-instructions and adjustment to tinnitus. *Journal of Psychosomatic Research*.

[39] Bayar N, Oğuztürk O, Koç C (2002). Minnesota multiphasic personality inventory profile of patients with subjective tinnitus. *Journal of Otolaryngology*.

[40] Marciano E, Carrabba L, Giannini P (2003). Psychiatric comorbidity in a population of outpatients affected by tinnitus. *International Journal of Audiology*.

